# Idiopathic Sclerosing Encapsulating Peritonitis in a Patient with Atypical Symptoms and Imaging Findings

**DOI:** 10.1155/2021/6695806

**Published:** 2021-04-07

**Authors:** Joseph Wetherell, Katherine Woolley, Rishi Chadha, Julia Kostka, Edin Adilovic, Pankaj Nepal

**Affiliations:** ^1^Frank H. Netter School of Medicine, Quinnipiac University, Hamden, CT, USA; ^2^Internal Medicine, St Vincent's Medical Center, Bridgeport, CT, USA

## Abstract

Sclerosing encapsulating peritonitis is a rare condition caused by a fibrotic membrane covering the small bowel which may lead to abdominal pain or obstruction. The cause may be primary and idiopathic or secondary to several diseases, treatments, and/or medications. The condition typically presents with bowel obstruction, and only one previous case has described ascites as the presenting sign. Sclerosing encapsulating peritonitis is typically diagnosed intraoperatively. We present a case of a patient who presented with atypical clinical symptoms including respiratory distress, recurrent abdominal ascites, and failure to thrive who was diagnosed nonoperatively.

## 1. Introduction

Sclerosing encapsulating peritonitis (SEP) or abdominal cocoon is a rare cause of abdominal pain and even small bowel obstruction (SBO). It is characterized by complete or partial encapsulation of the small bowel by a thick, fibrous membrane that resembles a sac [1–6]. Primarily, this condition is idiopathic, and the etiology is unknown [7]. It was first observed in 1907 by Owtschinnikow and was called chronica fibrosa incapsulata [1]. In 1978, this condition was described to also occur secondarily to peritoneal dialysis [2]. The rarity of this condition and the range of clinical features make this difficult to diagnose nonoperatively. Computed tomography (CT), in combination with clinical presentation, has been reported to be the most sensitive and specific nonoperative modality of diagnosis [8, 9]. The purpose of this case is to bring attention to unusual clinical features of SEP that have not previously been widely described.

## 2. Case

The patient was a 52-year-old male with a past medical history significant for end-stage renal disease (ESRD) on hemodialysis (HD), quadriplegia secondary to a gunshot wound (GSW), history of congestive hepatopathy with hemorrhagic ascites, heart failure with reduced ejection fraction (HFrEF) of 45%, and methicillin-resistant Staphylococcus aureus (MRSA) peritonitis who presented from home due to self-reported increased respiratory distress after missing his hemodialysis session the previous day. The patient had lost 20–30 lbs over about 3 months, with increasing fatigue, confusion, and fecal incontinence from decreased sphincter control. He was a tobacco user (1/2 pack per day), former excessive alcohol consumer, and former cocaine and marijuana user.

In the ED, the patient had normal and stable vital signs with labs significant for hemoglobin of 7.1, serum Cr of 3.4, and alkaline phosphatase of 944. On physical exam, the patient was seen to be cachectic with no evidence of scleral icterus. He had a markedly distended abdomen with a positive fluid wave and normoactive bowel sounds. He was admitted for failure to thrive from multiple chronic medical conditions and recent hospitalization a month prior at an outside hospital for MRSA peritonitis leading to deconditioning.

The patient had previously undergone liver biopsy and hepatic venous pressure gradient measurement that did not demonstrate any signs of cirrhosis or portal hypertension at that time. Paracentesis performed on hospital day 2 drained 5 liters of cloudy serosanguinous fluid with debris. Further analysis showed no malignant cells present, serum albumin-ascitic albumin gradient (SAAG) > 1.1, 60,000 RBCs, and 36,750 WBCs, demonstrating hemorrhagic ascites consistent with newly developed and diagnosed portal hypertension complicated by secondary bacterial peritonitis. The culture of collected fluid demonstrated MRSA growth in broth only. Infectious disease was consulted, who stated MRSA peritonitis was unlikely due to growth only in culture broth. They recommended acid-fast bacilli (AFB) stains, smears, and cultures to further investigate the source of infection. A noncontrast CT scan of the abdomen and pelvis was performed to determine a possible etiology for bacterial peritonitis, which did not reveal any such source. AFB workup proved to be negative as well.

Records obtained from previous hospitalizations at an outside hospital indicated that there was evidence of hepatic congestion and fibrosis without evidence of cirrhosis, with intent to undergo peritoneal biopsy that was deferred due to multiple comorbidities. Further analysis of the patient's abdominal CT scan demonstrated his significant ascites to be completely encapsulated and displaced anteriorly away from any visceral organs, consistent with diagnosis of SEP. This was said to be the cause of his abdominal ascites, with underlying malignancy unlikely. Consultation was placed to the general surgery service, who deemed the patient to be a poor surgical candidate for diagnostic and therapeutic laparotomy due to his significant comorbidities. The patient underwent a second paracentesis on hospital day 6, removing 2.5 liters of similar ascitic fluid compared to his prior study. It was recommended for the patient to undergo hepatic vein ultrasound for further investigation; however, the patient did not want any further treatment and instead wanted to be discharged home. The patient was found competent to make his own medical decisions and was discharged.

## 3. Images

**Figure 1:**
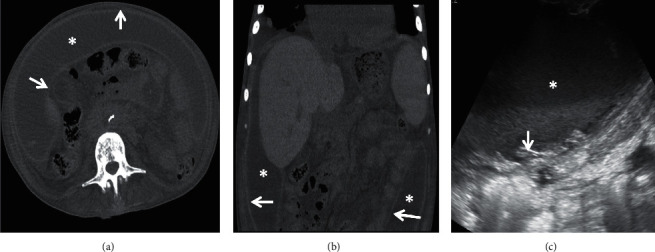
A 52-year-old male with sclerosing encapsulating peritonitis. (a) Axial noncontrast CT of the abdomen showing encapsulated ascites (∗) within the thickened layers of the peritoneum (arrows). The bowel loops are displaced centrally due to the mass effect. (b) Coronal noncontrast CT image shows the extent of the encapsulated fluid (∗) within the thickened peritoneum (arrows). (c) Grayscale ultrasound image acquired just prior to the paracentesis shows a low level of homogenous echotexture of the ascitic fluid (∗) and encapsulating thickened peritoneum (arrow).

The imaging findings of a 52-year-old male with sclerosing encapsulating peritonitis can be seen in Figure 1.

## 4. Discussion

Sclerosing encapsulating peritonitis, also known as abdominal cocoon syndrome, peritonitis chronica fibrosa incapsulata, encapsulating peritoneal sclerosis, and sclerosing peritonitis, is a relatively rare condition known to cause abdominal pain and either partial or complete SBO. It can be classified as either primary (idiopathic) or secondary, commonly caused by things such as chronic ambulatory peritoneal dialysis (CAPD), familial Mediterranean fever, TB, sarcoidosis, liver transplantation, cirrhosis, viral peritonitis, prior abdominal surgery, beta-blocker treatment with practolol, and VP shunting [7]. It was originally classified as occurring more frequently in women from tropical and subtropical regions, but as more cases have been reported, it does not appear limited to that group/region. Peritoneal dialysis has been shown to be associated with many types of peritonitis, including tuberculosis [10]. In one study in patients undergoing peritoneal dialysis (PD), incidence was reported to be 4.9 per 1000 person-years or 8.7 per 1000 person-years of PD [11]. The maximum risk of SEP was beyond 4 years of PD, at about one in 12 patients. Interestingly, our patient did not have any of these known risk factors and was on hemodialysis, never being on CAPD.

SEP has been described as having four distinct, progressive pathophysiologic stages as follows: a pre-sclerosing encapsulating peritonitis, an inflammatory phase, a progressive phase, and a fibrotic phase [2]. The fibrotic phase has been associated with the formation of a thick, fibrous plaque surrounding abdominal visceral organs and causing SBO. While this case does not demonstrate a patient with SBO, it remains possible that the ascites could be caused by a constricting force on the liver and/or portal vasculature. SEP is further classified by the contents of the membrane. Types 1 and 2 involve either part or complete intestine, and type 3 involves the small intestine, appendix, cecum, ascending colon, stomach, liver, and ovaries [12]. Interestingly, our patient's abdominal CT did not indicate any abdominal contents encased within the membrane, only ascitic fluid. This is a unique representation of SEP.

While classically reported to present with signs and symptoms of bowel obstruction, a recent report showed a diagnosis of SEP in an otherwise healthy female also presenting with massive abdominal distention with ascites [13]. This presentation is similar to our case. Our patient's clinical features included failure to thrive and ascites with no small bowel obstruction. Based on a search of the literature, this is the only other case reporting similar signs in a patient with SEP. This condition can be devastating and life threatening if not discovered, and its rarity adds to the diagnostic challenge. Thus, it remains critical that healthcare providers are aware of the spectrum of clinical signs and symptoms this condition may present with. Radiographic studies lend additional assistance in arriving at the diagnosis of SEP. Currently, CT imaging of the abdomen is the radiologic imaging of choice to make the diagnosis of SEP [9]. Specifically, noncontrast-enhanced CT has been shown to better depict membranous calcifications. Normally, the peritoneum is thin, smooth, and barely perceptible with discontinuous enhancement on CT imaging. Yet, in SEP, the peritoneum is markedly thickened, calcified, and with continuous enhancement. Additionally, the small bowel will be congregated centrally in the abdomen. This can be seen in Figures 1(a) and 1(b), with continuous enhancement of the peritoneum that encapsulates ascitic fluid in a horseshoe-like shape surrounding centrally located loops of the small bowel.

It is agreed upon that operative resection of the fibrous membrane is the definitive treatment. Operative management is preferred in patients with acute small bowel obstructions or if other organs are involved [13]. However, our patient was assumed to present in the earlier stages as he did not have an obstruction. Conservative management is an option for asymptomatic patients without obstruction, and steroids, tamoxifen, colchicine, azathioprine, and mycophenolate mofetil have been utilized [12, 13]. Tamoxifen specifically has been utilized in multiple other diseases of excessive fibrosis, such as retroperitoneal fibrosis, fibrosing mediastinitis, idiopathic sclerosing cervicitis, and desmoid tumors [14]. The likely mechanism of action for tamoxifen is the inhibition of fibroblast TGF-*β* production [15]. One recent case report of a 28-year-old male treated for 10 years with tamoxifen, along with 18 months of corticosteroids, demonstrated strong clinical improvement with little to no medication side effects; interestingly, CT findings of calcified peritoneal thickening remained unchanged over that time [16]. Our patient presented without obstruction and was deemed a poor surgical candidate, so this case was diagnosed noninvasively and is being actively observed. It is possible that our patient could be a candidate for nonsurgical medical management, such as with tamoxifen therapy, if he decides on continuing treatment in the future.

## 5. Conclusion

SEP is a rare condition that is difficult to diagnose. Our subject presented with unique clinical and radiologic features expanding on the wide range of presentations seen in SEP. Noninvasive diagnostic imaging is preferred for nonacute, poor surgical candidates, and abdominal CT remains critical. Conservative management is adequate in patients without acute symptoms. This case adds to the current literature regarding presentation of SEP, and clinicians should be aware that ascites and failure to thrive may be a sign of this devastating, rare condition.

## Data Availability

No data were used to support this study.
